# The Power of Personal Service

**Published:** 1919-05-10

**Authors:** 


The Hospital

The Workers' Newspaper of Administrative Medicine and Institutional Life, Administration, National Insurance and Health.

No. 1718, Vol. LXVI. SATURDAY, MAY 10, 1919. PRICE TWOPENCE

### THE POWER OF PERSONAL SERVICE.

Tuesday, May 6, was the anniversary of the death of King Edward VII. of blessed memory, whose heart was in the King's Fund from its inception to his death. On that day the twenty-second annual meeting of this Fund was held, when H.R.H. the Prince of Wales took the chair as its President for the first time. He spoke with admirable diction and attractive force, and won the hearts of all who heard him. It can be seen from the full report which we give of his speech, and of the present position of this Fund, with what remarkable completeness King Edward's hopes of the work it could accomplish have been fulfilled.

There are few, if any, fields of work which can bring greater uplifting of heart and joy in continuous fulfilment, not only to youth but to old age, not only to the humblest but to the highest in the land, than the purposeful continuous practice of personal service for the sick. Many, by the unfailing practice of such service, realise the privilege of habitually giving time each year to the service of others. Without such service from a multitude of voluntary workers it would have been impossible for the King's Hospital Fund to be what it is universally admitted to be to-day, *i.e.* the treasured means whereby financial strength and financial weakness, the richest and the poorest of the citizens, have gladly joined forces to make it strong enough to fulfil the purposes for which the Fund was originated. In fact the King's Hospital Fund has received at least half a million sterling from one donor, and regular gifts of 1s. a year from tens of thousands of poorer Londoners. The voluntary hospitals owe probably more to the King's Fund than to any other agency which has been established in the world's history in aid of the hospitals.

No doubt such service, originating in the Sovereign and spreading through all classes from the poorest to the wealthiest, has made the King's Fund and the League of Mercy the undoubted powers for good which they have been proved to be. Men of heart and untiring energy, who have and do continue to work for the King's Fund, because they believe in the principles upon which it is founded, have been known to declare that there are few things, if any, that have brought them greater happiness in their lives, and a larger realisation of the blessedness of human sympathy. Amongst them have been all types and classes of people, who rejoice at the personal service which it has been their privilege to render through this Fund.

The war has heightened the agony and suffering of mankind beyond everything history records. The terrible injuries which medical and surgical science had to face, and in God's mercy relieve, the work which devolved upon the sisters and nurses who gave of their best in their services to the wounded warrior, the call for the exercise of scientific and mechanical ability of the highest type, all the many problems which those responsible for treatment in the military hospitals had to solve, and the magnificent results attending the whole-hearted efforts of these noble men and women, have brought about an amount of team work in hospital treatment which cannot fail to revolutionise much which has long passed current in our hospitals. Already there is evidence that momentous changes may be anticipated in the near future. Those changes will involve increased expenditure in some directions, and diminution in cost in others. The claims of the men, who have saved the nation and Empire in this country, entitle them to obtain provision for hospital care and treatment, which will not only tend to their recovery, but provide for their transfer at the earliest possible moment to hospital camps in the country, where they will be retained until they are convalescent, and well capable of resuming their employment on their arrival at home.

The recent terribly fatal epidemics of pneumonia have demonstrated the need for radical changes in the organisation and treatment at present pursued in regard to these cases, and the substitution of paid medical service to a considerable and increasing extent, with a system of team work calculated to secure prompt diagnosis, treatment designed to promote shorter residence in the hospital, and transference by ambulance to the hospital camps to be provided outside the cities. The future therefore promises a field of extended interest to us all, with improved conditions for the individual patient, and better and juster treatment for the members of the great medical profession.

